# On evolutionary origin of cancer

**DOI:** 10.1186/1475-2867-5-5

**Published:** 2005-03-02

**Authors:** Anatoly V Lichtenstein

**Affiliations:** 1 Laboratory of Tumor Biochemistry, Cancer Research Center, Kashirskoye shosse 24, 115478 Moscow, Russia

## Abstract

**Background:**

The necessary and sufficient capabilities of cancer cell have been identified. Strikingly, this list does not include one that would seem to be a key property, namely the ability of cancer cells to kill their "host". This is believed to be a self-evident consequence of the other capabilities (e.g., metastasis), although the available evidence suggests a distinct killer function. Taking into account this unlisted property can significantly affect the current paradigm of carcinogenesis.

**Presentation of the hypothesis:**

On the assumption that killer function is a key capability of the cancer cell, it is suggested that cancer has evolved as a mechanism of negative selection of mutant alleles of vitally important genes present in population. Similarly to apoptosis, which is an altruistic suicidal act of a damaged cell, cancer is an altruistic suicidal act of an individual who carries dangerous alleles and presents a hazard for genetic stability of the population. From this point of view, apoptosis is not a protection means against cancer as generally believed, but rather they are the first and second lines of defense against genome instability, respectively.

**Testing the hypothesis:**

The modern DNA array technology is capable of revealing gene expression profiles responsible for killer function of cancer cell as well as those specific targets in the body that are most strongly affected by the tumor growth.

**Implications of the hypothesis:**

This hypothesis suggests new avenues of cancer research as well as principally new therapeutic strategies.

## Background

We are now witnessing a post-genomic era of cancer research. Thousands of papers are devoted to discovering molecular mechanisms of this extremely complicated program, the latter term being understood as a prescribed sequence of events with an inevitable result. In almost all studies cancer is considered as a given entity with no attempts made, to my knowledge, to search into its evolutionary origin. However, cancer almost certainly fulfills some evolutionary tasks.

First, although cancer is usually mentioned as a representative of a large group of age-related diseases, it principally differs from all the others. Cardiovascular diseases, diabetes, Alzheimer's disease, and many other diseases are manifestations of the "loss-of-function" phenotype due to degeneration and/or death of corresponding cells. Cancer, on the contrary, is an active "gain-of-function" process. Cancer cells acquire numerous new functions, including the unique abilities to adapt to a changing environment and to dodge the blows of the body's protective means, as well as a striking capacity to recruit surrounding normal tissues. The tumor transforms its natural enemies (normal cells) into unnatural allies, being able to grow only having such a paradoxical support [1-15]. The functional relations between normal and cancer cells are so diverse and the tumor structure is so complex and hierarchical that a growing tumor is sometimes viewed as a special developing "organ" [2,16]. Such an "organ" must have serious evolutionary grounds to have evolved.

Second, cancer is an inevitable attribute of the animal world. It hits every species despite the fact that there are huge differences among them in the intracellular machinery, organization of signal pathways and, accordingly, in anticancer defense (or, transformation resistance, see below). In no case, however, is the anticancer defense of a species high enough to exclude completely this illness, despite presumably high cellular plasticity in this respect. This fact would indicate some evolutionary advantages of possessing such a trait. It seems likely that anticancer defense of a species is counterbalanced by opposing forces acting on the population level. In other words, cancer incidence among a species is presumably maintained at certain favorable level, which is coupled in each particular case with an evolutionary adaptation of the intracellular machinery.

In conclusion, evolutionary grounds for cancer seem to be evident; it is the explanation of its expediency which presents difficulties. A clue to this problem would be the fact that cancer cell possesses, apart from the well known necessary and sufficient capabilities [17], a killer function directed against the host. Strikingly, this evident capability that seems to be a key property of the tumor cell does not attract the attention it deserves. It is believed to be a self-evident consequence of the other traits that constitute the malignant phenotype, although the available evidence would rather suggest that killer function is a distinct capability. Taking into account this overlooked property, one can view cancer as a suicidal act of an individual, since the inevitable outcome of this illness is demise (if treatment is absent or delayed). By analogy with apoptosis, which has evolved as altruistic suicidal act of a damaged cell [18] that otherwise would threaten the genetic stability of the cell population, cancer might be viewed as an individual suicidal act that brings some benefits to the population.

## Presentation of the hypothesis

### Killer function is a key capability of the cancer cell

Carcinogenesis is a multistage process of accumulation of gene defects that determine the characteristic traits of the cancer cell: self-sufficiency in growth signals, insensitivity to anti-growth and pro-apoptosis signals, limitless replicative potential, sustained angiogenesis, tissue invasion, and metastasis [17]. These acquired capabilities determine the malignant phenotype of a cell but do not explain the clinical manifestations of cancer. Indeed, it seems astonishing that the human body, which consists of ca. 10^14 ^cells, can not endure a relatively small fraction of cancer cells (0.01 – 0.1% of the total), this burden often being incompatible with life. Rarely, the immediate cause of death is evident (brain compression, bleeding, perforation of the intestine), but in general it remains obscure. This suggests a deadly influence of cancer upon the body through some unknown mechanisms. Although each of many different forms of cancer has clinical peculiarities, the overall course of the illness and the final result are always the same. So, the notion that the tumor cell, regardless of origin, possesses a special killer function is a statement of an obvious fact. What is surprising in this regard is that no room has been allocated to it in the existing paradigm of carcinogenesis.

Paraneoplastic syndromes present evidence that tumors may affect normal tissues remote from the primary site. These syndromes are extremely diverse and affect almost all organs and tissues [19]. The most frequent clinical manifestations are cachexia, anorexia, nausea, neuropathy, retinopathy, general sickness, and malfunctions of many body systems. A long time (16 to 20 months) before cancer is diagnosed, some patients show body weight loss, which is indicative that even at early stages tumors may have a generalized effect upon the body, which increases progressively with tumor growth [20]. Since, however, pronounced cachexia (a loss of more than 5% body weight) occurs in about a third of patients and becomes the cause of death in only 20% of the cases [21], it is clear that tumor cells have other, yet unrecognized means of killing the body.

It remains unknown whether the paraneoplastic syndromes are direct manifestations of the killer function itself or they are mere side effects of tumor growth, such as autoimmune diseases. It is noteworthy that cancer is not always accompanied by paraneoplastic syndromes [22], yet its killer function never fails. On the other hand, the most effective treatment of paraneoplastic syndromes is specific cancer therapy, while the reverse approach, a symptomatic treatment targeted at particular manifestations of tumor growth, rarely gives positive results and never offers a radical cure. This fact suggests that most of the mentioned effects, justifying their name of *para*neoplastic, take place not within the killer function pathway but somewhere aside. Because of this, the term killer function will be used here, without going into its mechanism, as indicating the obvious capability of cancer cells to kill the body.

The killer capability is crucial to the achievement of the final goal, body demise, whereas all the other, promoting proliferation and spread of killer cells are *de facto *only accessory. It seems to be a distinct capability of cancer cells, rather than a derivative of other capabilities, such as uncontrolled proliferation and metastasizing. It is unlikely that active proliferation can by itself exert such a deleterious effect since dozens of billions of cells divide daily in the human body, which is many-fold greater than the proliferation pool of the biggest tumor. Even the metastases, these relatively small foci of ectopic proliferation, can not account by themselves for the inevitable demise. The killer function is also a universal property of the cancer cell since without treatment the lethal outcome is inevitable no matter what is the type of tumor, its ability to develop metastases, recur, induce cachexia, or affect biochemical indices. Finally, this property is specific to the cancer cell, as in normal cell physiology there are no examples of such activity.

The killer function seems radically different from all other acquired capabilities in that it apparently gives no selective advantage to the cancer cell. On the contrary, for the latter, as a part of the body, acquiring such a function is the same as committing suicide. This changes radically the understanding of the role of cancer cells: they can be regarded not as selfish "cheats" [23], which propagate at the expense of all others, but rather as altruists which sacrifice themselves and the whole body for the sake of some higher (apparently population) benefits, as suggested in the recent hypothesis of phenoptosis [24].

### Mutations as death program trigger

**Figure 1:**
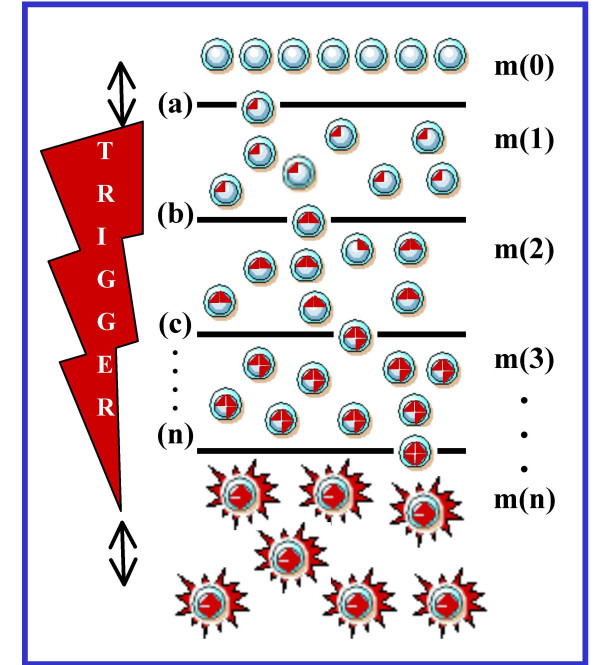
**Step-wise accumulation of specific gene defects. **Mutations (red triangles) trigger the built-in cell killer function (red asterisks). m(0), normal cells; m(1), m(2), m(3), m(n), mutant cells with 1, 2, 3, and *n *defects in cancer-related genes, respectively. (a), (b), (c), (n), selection "sieves" which determine the stages of transformation. The trigger "count-down" is shown on the left. Double-headed arrows indicate that transformation and trigger steps are amenable to species- and tissue-specific variations (see text).

As cancer cells do not acquire selective advantages during realization of their killer function, it seems unlikely that the latter is created each time *de novo *in the same way as the other properties are, namely in the way of numerous step-by-step cycles of mutation-selection. Rather, the cells possess a built-in and ready-for-use program of deadly events, which, just like apoptosis, is launched under certain conditions and then functions automatically. In such a case, mutations of cancer-related genes are not only transformation steps, as generally considered, but also a trigger countdown mechanism that activates the death program directed against the "host". This dual activity leads to appearance of an expanding clone of killer cells progressively strengthening their effect upon the organism (Fig. 1).

A specific feature of the genetic defects underlying the tumor phenotype is that they do not decrease cell viability (basic cellular functions, in contrast to specialized, are even enhanced). In a certain sense, these are the defects of not the cell *per se *but rather of the cell/organism "interface" that mediates subordination of cell functions to the common interests. Under normal conditions, numerous signal pathways tie the cell to the tissue homeostasis mechanism, whereas their defects are nothing but a step-wise liberation of the cell from its "fetters". The number of liberating mutations necessary for cell transformation to occur ranges from 4 to 12 [25,26], this value being a quantitative measure of the cell "transformation resistance". If one can compare the cancer cell with an explosion device aimed at destroying the body, then mutagenesis serves there the function of a trigger countdown clock with 4–12 intermediate positions (Fig. 1).

### Peto paradox

Cell transformation is an extremely rare event because it requires the coincidence in a single cell of several very improbable events [26-28]. However, the life-span risk of human cancer is high (ca. 20%) because of a huge number of cells in the body (ca. 10^14^) and large longevity. Indeed, no matter how unlikely is the event by itself it has a real chance to occur under such conditions. If so, one might suggest that animals with a small body weight and short life-span (e.g., rodents) should not suffer from cancer at all, while big animals (whales) should get cancer in their mothers' wombs. Reality, however, does not follow this theory's predictions (the well known Peto paradox [29]). All animals regardless of body weight and longevity suffer from cancer, but, on the other hand, cancer incidence does not threaten the species existence.

The Peto paradox is explained by various transformation resistance of cells from different species [23,30]. This may be due to (i) different efficiency of DNA repair systems, (ii) difference among species in the degree of selective advantages acquired by the cell from similar mutations [30], (iii) different setting up of the trigger (e.g., more signal pathways have to be damaged to transform human than mouse fibroblasts [31]). The trigger is probably most reliable (i.e., has a greatest number of intermediate positions) in whale cells and least reliable in mouse cells.

The Peto paradox can apparently be applied to body tissues as well [23]. Indeed, cancer develops in all human tissues, which differ greatly in the number of cells as well as their proliferation activity. Just as in the case of interspecies variability, it can be assumed that cells of different tissues have different transformation resistance [32,33]. The presented examples suggest that the transformation resistance of cells from different tissues and species underwent evolutionary adaptation to the selective pressure exerted by tumor growth [23].

### Cancer is a local manifestation of generalized mutagenesis

Most patients develop only one tumor, which presents carcinogenesis as allegedly a local process. Experiments with exposure of animal skin to chemical carcinogens as well as cases of occupational cancer, which demonstrate clearly the link between site of exposure and tumor localization, support this notion. Without questioning the significance of such observations, they seem to have an exceptional and limited character (see below).

In fact, mutagenesis is intimately related to metabolism and is therefore omnipresent: every day in each cell many thousands of DNA lesions occur due to replication and repair errors, spontaneous depurination, methylcytosine deamination, reactive oxygen species attacks, and telomere shortening. This list should perhaps be extended to include the mutagenic effect of apoptosis resulting from uptake by phagocytosis of DNA from dead cells [34]. Because of imperfect repair of DNA damage, the mutation rate is estimated to vary in the range of 10^-4^–10^-8 ^per gene per cell division [26,27]; mutations occur in all tissues and increase with age [10,27,35,36]. Calculations show, for instance, that by the age of 65 over 10^5 ^mutations accumulate in the normal stem cell of human colonic crypt [28].

When a tumor nodule appears in the body, it seems to be only the tip of an iceberg, maturing in the body for decades and consisting of a multitude of damaged cells at different stages of transformation. This assumption is supported by the clinical experience showing that overt symptoms of the disease are always preceded by precancerous lesions, such as hyperplasia, metaplasia, and dysplasia. This idea finds further development in the concept of "field cancerization", i.e., large (more than 7 cm in diameter) and surrounding the tumor "patches" of damaged cells, recognized on the basis of mutations in *TP53*, but remaining undetectable by routine diagnostic techniques [37].

Similar conclusions can be drawn from a notion of mutation as a random and rare event and carcinogenesis as accumulation of genetic defects. Mutagenesis can be described as the process both extensive (measured by number of affected cells) and intensive (measured by number of mutations per individual cell). Evidently, these parameters are positively correlated with each other: the wider the damaged zone, the deeper the damage of individual cells. The reverse is also true: the deeper the damage of the individual, most "advanced" cells, the wider the lesion area (this means that the very fact of a tumor appearance is, in general, the sign of a significant mutagenic lesion). In other words, appearance of the cell having a complete set of mutations (i.e., cancer cell) is accompanied by formation of a large pool of precancerous cells.

**Figure 2:**
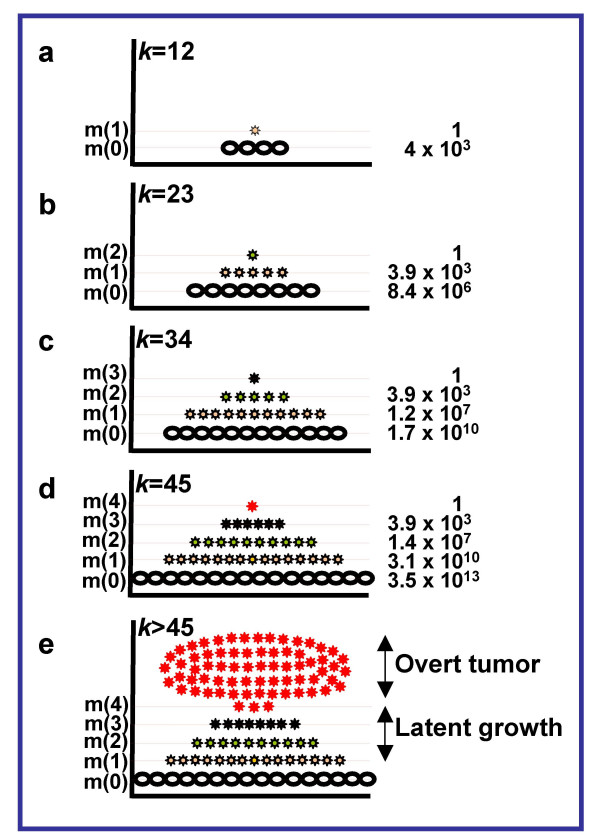
**Maturation of cancer "mushroom" in exponentially growing tissue. ***k*, cycles of cell exponential growth; *u*, 4 × 10^-5^. a – e, stages of carcinogenesis. m(0), normal cells; m(1), m(2), m(3), and m(4), mutant cells with 1, 2, 3, and 4 mutations in cancer-related genes, respectively. Cell numbers in each layer are indicated on the right (see text).

The simple model of accumulation of mutant cells (i.e., cells with 1–4 mutations, the latter being the arbitrary transformation threshold) in an exponentially growing cell population is shown in Fig. 2. With time mutant cells inevitably appear [28], since mutagenic load is increasing and the repair systems become less efficient [35]. The first to appear and start to grow is a layer of cells with one defect, then a layer of cells with two defects, and so on. Each subsequent cell layer grows quicker than the preceding one because each new mutation confers a selective advantage to affected cells [38]. Additional momentum is conferred to the entire process by acquisition of chromosomal instability [39] or a mutator phenotype [26]. At each stage of carcinogenesis, transition from quantity (of damaged cells) to quality (a cell with a new mutation) takes place, the latter having a chance to appear only from a large enough pool of its predecessors. On the whole, maturation of a tumor looks like a "pyramid" growing until a completely transformed cell appears at its top. The latter gives rise to the overt tumor. The overall process may be symbolized by a "mushroom", in which the "stem" and "cap" are the latent and overt stages, respectively (Fig. 2).

It follows from the model of multistage carcinogenesis [23] that in an exponentially growing tissue the number of mutant cells is approximated by the formula



where  is the mean number of cells with *m *mutations after *k *cell generations and *u *is the mutation rate. If we assume that the body is composed of 3.5 × 10^13 ^cells (i.e., 2^45^, *k *= 45), and *u *is 4 × 10^-5 ^(for a single gene *u *is 4 × 10^-7^, thus if there are one hundred cancer-related genes *u *becomes 4 × 10^-5 ^[40]), then at the moment of appearance in the body of the cell with 4 mutations (a tumor founder), there will be ~3.9 × 10^3 ^cells with 3 mutations, ~1.4 × 10^7 ^cells with 2 mutations, and ~3.1 × 10^10 ^cells with 1 mutation (Fig. 2d). In other words, in the human body one cell from every thousand (0.1%) will be mutated at least in one of the cancer-related genes. In reality, mutation frequency should be much higher because cell death occurs during developmental growth so that replenishing cell divisions must increase *k *value significantly. This calculation, though very approximate, agrees with experimental data [27] and can give an idea of the magnitude of the mutation frequency (see also [28]). They might indicate that cancer appears as mutation frequency reaches a certain security threshold. On the other hand, this calculation showing that the human body is flooded with mutant cells prior to tumor appearance points to the validity of chemoprevention as an essential approach to controlling cancer [41].

### Cancer as a mechanism of negative selection of mutant alleles

As discussed above, the size of the fraction of pre-malignant cells is dependent on trigger tuning – the more mutations needed for a malignant transformation, the bigger the pool of damaged cells. Hence, the first completely transformed cell plays *de facto *a triple role: (i) a *sensor* for general mutagenesis (since its "trigger clock" counts down in accordance with accumulation of mutant cells in the body), (ii) an *executioner *that unleashes a built-in death program after mutagenesis exceeds some threshold level, and (iii) a *founder* of a clone of killer cells. Any stem cell in an organism can apparently play such a triple role, thus ensuring reliability of the mechanism.

Evolutionary conservation of the death program prompts us to look for an explanation for its appearance. It may consist of the counter-selection of genetically defective individuals. As a matter of fact, if an arising tumor manifests significant whole body mutagenesis, then germ cells are most probably not an exception: a similarity of spontaneous germinal and somatic cell mutation rates was shown for human cells *in vitro *[42]. High levels of somatic mutation seem to be a direct reflection of the germ line mutation rate selected over evolutionary time [35]. Due to its prolonged solitary existence and relative lack of protective, repair and self-destruction mechanisms, sperm cells may be even more susceptible to genetic damage than somatic cells [27,43,44]. Besides, some gene mutations are paradoxically enriched because they confer a selective advantage to the spermatogonial cells in which they arise [45,46]. In conclusion, germ cells are apparently not protected from mutagenesis more reliably than somatic cells are. If so, one can hypothesize that the killer program (i.e., cancer) is unleashed in a somatic cell when its vital ("cancer-related") genes are damaged to such an extent that testifies to intolerably high mutation frequency in body tissues and, most importantly, in germ cells ("in mutant soma a mutant semen" principle). This may endanger the genetic stability of the population. Therefore, if at the cellular level cancer cells look like aggressive "cheats" [23], at the body level the process may be an altruistic suicide to remove mutant alleles from the genetic pool.

While in the case of sporadic tumors the notion that cancer is a local manifestation of generalized mutagenesis is only a more or less validated assumption, it is a truism in the case of hereditary tumors [47]. Carcinogenesis in such an individual has significant "odds" compared to wild-type individuals [11] because all his cells are mutant (the trigger, in other words, has been moved one position ahead from the very outset). The association between the evident threat of germinal mutations to the population, on one hand, and their extremely high cancer risk, on the other, seems not to be by chance. The life-span risk of getting cancer for persons with germinal mutations in the suppressor genes reaches 50–80%, and their tumors, often multiple, appear at a reproductive age [11]. Owing to an extremely strong selection pressure, the alleles that predispose to cancer are very rare (ca. 1:1000 or less), suggesting that the mechanism is efficient. The most convincing argument for the hypothesis that malignant tumors have an altruistic function comes from hereditary forms of cancer in which the association "mutant semen in a mutant soma" is most evident. The altruism here is that the carriers of mutant alleles die at a reproductive age. It is germinal mutations arising in a population with a certain frequency that could have been the driving force for cancer evolution.

It is evident that the greater the number of pre-cancerous stages, the more strongly the individual is protected against cancer. A computational model of cancer progression was elaborated recently to show that the appearance of an extra stage and the additional buffering, which arises as a result, reduce the impact of any single hereditary mutation and therefore allow the accumulation of more nonlethal mutations in the population [48]. Because natural selection cannot purge mutations that are mostly hidden by robust pathways, mutations will continue to accumulate until their consequences become sufficiently deleterious that they are balanced by natural selection. An additional protection from cancer by extra stages thereby leads to the evolution of partially decreased cancer mortality and significantly increased genetic variability in the population as a whole [49]. This point of view is in accordance with the assumption that if such purging mechanism as cancer was absent, deleterious mutations would be widespread in the population.

There are many germinal mutations with phenotypic expression but just a few of them (affecting a small group of ~50 genes [50,51]) are linked with hereditary cancer. Probably, only mutations in key genes that present the greatest threat to the population are prevented by this mechanism from being spread. Many other germinal mutations are unable to pass the "sieve" of embryonic development because they induce early abortions [27]. There are a number of barriers to prevent spreading of mutant alleles in a population, and cancer seems to be only one of them.

A favorable trait is retained only if it manifests itself during the reproductive period. So, the fact that cancer is predominantly a disease of the elderly would seem to be at conflict with a supposition of its evolutionary significance. This is perhaps an apparent conflict: the incidence of cancer among the young may be relatively low exactly because of the efficient selection against the adverse alleles that constantly appear in the population and exist as an inevitable background. As regards the high incidence of cancer at the old age, which seems to have no evolutionary significance, this can be explained from the viewpoint of the evolutionary theory of antagonistic pleiotropy [52]. A genetic program that has played a positive role in youth continues to be active in the older age, despite its possible counter-productive effects, simply "from force of inertia" because no correction mechanisms are available [53]. This, together with an increased mutation and weaker repair, results in the exponential growth of cancer incidence in old age [10,35].

Germinal mutations in functionally important genes are a strong stimulus for development of a mechanism to prevent their spread in the population. Apart from these highly penetrant rare alleles with a strong hereditary predisposition to cancer, which are merely eradicated from the population, there are many alleles that determine a weak predisposition [49] (their cumulative effect can be very significant [11,54,55]). The frequency of such alleles in a population seems to be negatively correlated with the cancer risk they contribute.

There is another apparent contradiction between the thesis that tumor appearance manifests generalized mutagenesis and the numerous cases when a tumor is clearly linked with a local exposure – inflammation, bacterial infection, or UV-irradiation. The occupational cancer, on one hand, and experimentally induced tumors in animals, on the other hand, makes this association unquestionable. This linkage is probably a side effect of the evolutionary mechanism described here, in which the tumor cell plays a dual role, the mutagenesis sensor and the death program executor. In such a mechanism, a local fluctuation can unleash a process even when the overall mutation level is low (similarly, a sensor designed to respond to whole system temperature is activated by a local heating).

In carcinogenesis, the hypothetical sensor/executor functions under the conditions of continuous interference and noise, which are generated by numerous external and internal locally acting mutagens. From this, many false actions ensue. If one takes the hypothesis that cancer is a means of protection, one must admit that in humans this mechanism is hyperactive and operates beyond the originally set objectives. Both the high cancer incidence in old age and the multitude of cancer cases resulting from local exposure to carcinogens are examples of this hyperactivity that obscure the true evolutionary nature of the phenomenon.

Evolution hypotheses that attempt at explaining the appearance of cooperation and altruistic behavior are based on the ideas of kin or group selection [56,57]. Their weakness is sometimes seen as an inequality between gross individual losses and relatively small population benefits, thereby questioning the validity of the proposed mechanisms. However, in the case of germinal mutations affecting important genes, the threat of genetic imbalance in the population is perhaps so high (see [48]) that it justifies the individual losses due to such a protection means as cancer. Here again one can perceive an analogy with cell suicide, which is likewise hyperactive in "forestalling" the potentially hazardous consequences of genetic defects (a weakening of such a preventive defense is undesirable [58]).

Maintaining DNA integrity is one of the main priorities of living organisms. Depending on the extent of DNA damage, three outcomes are possible: (i) small damage induces repair which restores the initial state; (ii) strong damage launches apoptosis thus preventing cell-to-cell transfer of damaged DNA; (iii) accumulating lesions, when apoptosis is impossible, trigger cancer thus preventing individual-to-individual transfer of damaged alleles of vitally important genes. From this point of view, apoptosis is not a protection means against cancer as generally believed, but rather they are the first and second lines of defense against genome instability, respectively.

## Testing the hypothesis

A striking discovery was made recently that a tumor can survive, propagate, and spread in the body only through the unnatural help coming from normal tissues [6,13,59]. One more step further in elucidation of tumor-host relationships is yet to be made, namely, a discovery of a mechanism of killer function. Although it is this feature that imparts so much significance to malignant growth, the current paradigm of carcinogenesis does not envisage the killer function as some special property; as a result, this function does not attract the attention it deserves. Meanwhile, there are possibilities to unveil the mechanism of tumor malignancy at present. The modern DNA array technology is capable of revealing gene expression profiles responsible for killer function. This can be done by comparison of (i) malignant tumors having different expression of this trait and (ii) benign and malignant tumors.

Besides, identification of genes responsible for killer function is to be supplemented, using the same technology, with serial analyses of expression profiles of various organs and tissues of tumor-bearing animals at various stages of tumor progression. This can help to identify those specific targets within the body that are most strongly affected by the tumor growth.

## Implications of the hypothesis

Recognition of killer function as a crucial capability of cancer cells suggests not only new avenues of cancer research (see above), but also principally new therapeutic strategy. Achievement of better understanding of mechanisms of body death may help to pinpoint new targets for therapy, such as some factors presumably emitted by cancer cells (unusual cytokines, for example), that exert the deadly effect. These factors are likely to be cancer-specific, so their elimination would not entail severe side effects. The present day cancer therapy is based, without much success, on the imperative to "exterminate the evil" (i.e., the cancer cells). The essence of an alternative strategy which may turn out to be more effective is not to kill the cancer cells, but to neutralize them.

## Competing interests

The author(s) declare that they have no competing interests.
